# Radiation dose absorbed by patients and professionals in an intensive care unit

**DOI:** 10.1186/2197-425X-3-S1-A734

**Published:** 2015-10-01

**Authors:** R Fernández Fernández, M Moreno-Torres, M Muñoz Garach, D Guirado Llorente, MI Núñez Torres, O Moreno Romero, L Peñas Maldonado

**Affiliations:** Hospital Universitario San Cecilio, Unidad Cuidados Intensivos, Granada, Spain; Hospital Universitario San Cecilio, Servicio Radiofísica Hospitalaria, Granada, Spain; Universidad Granada, Dpto de Radiología y Medicina Física, Granada, Spain

## Introduction

Chest radiographs are the most frequent diagnostic tests that are performed with X-ray in an intensive care unit (ICU). The riskthatthestaffperceives from these examinations produces the evictionof the room during the examination to avoid potential exposure, with a consequent impairment of the quality of care offered to patients. We do not knowhow widespread this practiceactually is.

## Objectives

Determine the dose absorbed by staff and patients in an ICU room due to daily X-ray studies, and to record the direct exposure of patients undergoing X-ray examinations.

## Methods

The work has been conducted in our ICU, which has 18 beds. The X-ray device used for the examinations is a ´RADIOLOGIA´ Transportix 32 C 6-28159, a mobile X-ray equipment with the standard features required in this service. The Dose Area Product (DAP) was measured at each examination by using a calibrated Gammex DAP-841S transmission chamber installed on the diaphragm of the X-ray equipment. We used four TLD dosimeters (ofthose commonly used inpersonal dosimetry), calibrated and read by the Spanish National Center of Dosimetry (CND) and consist of four LiF:Mg, Ti detectors sandwiched between different filters and integrated in a Vinten 860-N52 board. The dosimeterswere placed in the following way: three in fixed positions (one each on the inner lintel above the two exit doors and one attached to the center of the ceiling) and one that was placed on the bed adjacent to the patient at each X-ray examination. A total of 132 studies have been collected from March to April 2014, 110 of them have been chest examinations carried out with a tube tension of 80 ± 5 KV (mean ± standard deviation). The data have been stored in a database anonymized and disaggregated for further statistical analysis.

## Results

The value given by the DAP is 94 ± 17 mGy cm^2^. This value is well below the lower limit recommended by different agencies and committees [1],[2]. Based on the TLD readings provided by the CND and taking account of the error margin (0.0 ± 0.1 mSv), the annual dose extrapolated from the measurements was less than 0.6 mSv, below the natural background doses in our area.

## Conclusions

1) The vast majority of tests performed are chest radiography.

2) The low value of DAP suggest that a good operation is being kept.

3) There is not scattered radiation in our ICU. The result is near 0.

4) There is no risk to other patients.

5) Healthcare professionals working in the ICU can be reassured that it is not necessary to leave the room while X-ray exams are conducted.

6)These data may be useful, most likely, to other ICUs, since there is a fear that the scattered radiation is widespread
